# Novel subdomains of the mouse olfactory bulb defined by molecular heterogeneity in the nascent external plexiform and glomerular layers

**DOI:** 10.1186/1471-213X-7-48

**Published:** 2007-05-16

**Authors:** Eric O Williams, Yuanyuan Xiao, Heather M Sickles, Paul Shafer, Golan Yona, Jean YH Yang, David M Lin

**Affiliations:** 1 Department of Biomedical Sciences, Cornell University, Ithaca, NY, USA; 2 Department of Biostatistics, University of California, San Francisco, San Francisco, CA, USA; 3 Department of Computer Science, Cornell University, Ithaca, NY, USA; 4 Department of Computer Science, Technion Institute, Israel; 5 School of Mathematics and Statistics, University of Sydney, Sydney, Australia

## Abstract

**Background:**

In the mouse olfactory system, the role of the olfactory bulb in guiding olfactory sensory neuron (OSN) axons to their targets is poorly understood. What cell types within the bulb are necessary for targeting is unknown. What genes are important for this process is also unknown. Although projection neurons are not required, other cell-types within the external plexiform and glomerular layers also form synapses with OSNs. We hypothesized that these cells are important for targeting, and express spatially differentially expressed guidance cues that act to guide OSN axons within the bulb.

**Results:**

We used laser microdissection and microarray analysis to find genes that are differentially expressed along the dorsal-ventral, medial-lateral, and anterior-posterior axes of the bulb. The expression patterns of these genes divide the bulb into previously unrecognized subdomains. Interestingly, some genes are expressed in both the medial and lateral bulb, showing for the first time the existence of symmetric expression along this axis. We use a regeneration paradigm to show that several of these genes are altered in expression in response to deafferentation, consistent with the interpretation that they are expressed in cells that interact with OSNs.

**Conclusion:**

We demonstrate that the nascent external plexiform and glomerular layers of the bulb can be divided into multiple domains based on the expression of these genes, several of which are known to function in axon guidance, synaptogenesis, and angiogenesis. These genes represent candidate guidance cues that may act to guide OSN axons within the bulb during targeting.

## Background

In the mouse olfactory system, each olfactory sensory neuron (OSN) extends an axon from the epithelium to the olfactory bulb to form synapses within structures called glomeruli [1]. The pattern of glomerular convergence is remarkably stereotyped [2] and is strongly dependent upon the specific odorant receptor expressed by the OSN [3,4]. But despite the number of cues that have been found that are expressed by OSNs or along the path traveled by OSNs to the bulb, surprisingly little is known about the role of the olfactory bulb itself in this process [5]. Transplantation and bulbectomy studies have observed the formation of glomerular-like structures by OSNs within cortical tissue [6]. Genetic ablation of mitral and tufted projection neurons showed that topographically correct convergence by P2-expressing OSNs still occurred in the absence of these target cells [7]. Disruption of the laminar nature of the mitral layer also did not affect convergence by P2-expressing OSNs [8]. These results have suggested that the olfactory bulb plays a minimal role, if any, in targeting [9,10].

On the other hand, studies ablating most of the bulb, as opposed to eliminating only projection neurons, have observed topographically incorrect convergence by P2-expressing OSNs, suggesting a role for the bulb in determining the ultimate location of OSNs [11]. Tracing studies have long suggested the existence of zone-to-zone mapping of OSNs onto corresponding domains within the bulb [12]. In *Drosophila*, *Dscam *expression in the antennal lobe is critical for proper olfactory receptor neuron targeting [13], providing the first data that the target structure is important for convergence. These experiments in *Drosophila *suggest that the mammalian bulb may also provide guidance information to OSNs [14]. Part of the uncertainty surrounding the role of the bulb in guidance is that it is unclear what other bulbar cells in addition to mitral and tufted projection neurons could provide targeting information. While radial glia have long been suggested as one possibility [7,15], cells in the external plexiform (EPL) and glomerular (GL) layers also possess many attractive characteristics. First, in addition to projection neurons, other cells in the EPL and GL form synapses with OSNs [1]. Second, recent studies point to a diversity of cell-types within these layers [16], and electrophysiological studies have shown the presence of different types of interneurons within the EPL [17]. Third, some glomerular cell populations migrate into the bulb much earlier than previously thought [18]. And finally, it has been shown that external tufted cells indirectly connect glomeruli that are innervated by OSNs expressing a common receptor [19]. Collectively, these data suggest that little is known about the different sub-types and developmental profiles of these cells. In one model, a subset of these EPL and/or GL cells could provide guidance information to OSNs. If this hypothesis is correct, then it should be possible to identify genes expressed by cells within these layers that are important for guidance and targeting.

Here we have undertaken a high resolution screen for genes that spatially define distinct regions of the EPL and GL. We used laser microdissection (LMD) coupled with RNA amplification, microarrays, bioinformatics, and *in situ *hybridization to search for genes that are differentially expressed by cells within these layers. We identified 23 such cDNAs and show that the superficial layers of the bulb can be subdivided into large regions based upon the various expression patterns. The patterns demonstrate the existence of gradients, localized expression domains, and medial-lateral symmetry within the olfactory bulb. Moreover, these patterns are maintained in cells that do not express *Tbr1*, consistent with the interpretation that they are expressed in non-projection neurons. We further show, using a chemical ablation paradigm, evidence consistent with the interpretation that these genes are expressed in cells that interact with OSNs.

## Results

### Spatial reconstruction of gene expression using laser microdissection

**Figure 1:**
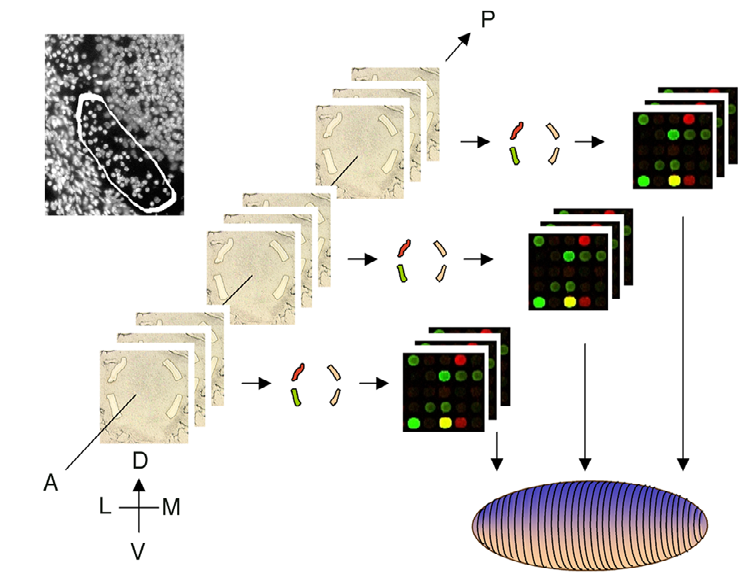
**Spatial reconstruction of gene expression using laser microdissection. **Above left – DAPI stain. Circled area indicates approximate size and cell number in each LMD sample. Left – nine sections were used for LMD that spanned the anterior, middle, and posterior bulb. Middle – four LMD samples were isolated from each section, sampling the DM, DL, VM, and VL aspects of the nascent EPL/GL. Right – RNA from each LMD sample was amplified and applied to microarrays. Bottom – data from the 36 arrays was synthesized to generate a three-dimensional statistical estimate of gene expression within the bulb for each gene on the array.

We performed our screen at embryonic day 17.5 (E17.5), as this is the first time that convergence is observed *in vivo *[20]. As the EPL and GL are not distinguishable at this stage [21] our samples contained cells from both developing layers (Fig. 1). We will refer to the regions where these samples were taken as the nascent EPL/GL. Only sections up to the front of the accessory olfactory bulb were used. These ~ 36 coronal sections were binned into three groups corresponding to anterior, middle, and posterior bulb (Fig. 1). As no prior genes have been identified as being spatially differentially expressed within the nascent EPL/GL, the size of each LMD sample, orientation, and location was chosen based on the ability to easily separate individual LMD samples. We also considered the known pattern of glomerular innervation by OSNs, which is shifted ~ 45° from vertical. We sampled cells from the dorsolateral (DL), dorsomedial (DM), ventrolateral (VL), and ventromedial (VM) nascent EPL/GL within each section (Fig. 1). Three sections were chosen from each group and four LMD isolates were obtained per section for a total of 36 isolates. RNA was isolated from the ~ 30–50 cells within each sample, amplified, and applied to microarrays [see Methods].

We synthesized the pool of microarray data into a three-dimensional representation of gene expression within the bulb [see Methods and Additional File 1]. As a result, all 33,000 cDNAs on the array could be queried to identify those whose expression patterns indicate differential expression within the bulb. For example, we examined whether or not expression for any given gene was higher or lower in the VM aspect relative to the remainder of the bulb. Genes were grouped into four categories [see Additional File 1] and the top 12 from each were tested by *in situ *hybridization. Of the 48 cDNAs, nine did not have any detectable signal. Of the remaining 39, 20 were spatially differentially expressed (51%). In one category (VL/DL), 83% were spatially differentially expressed. These results speak to the sensitivity of our analysis, as all samples differ essentially only by position. We also examined a small subset of genes chosen based on annotation. These genes, which were all ranked within the top 50, were selected because they correspond to known, transmembrane-bound molecules. As such, they would be more likely to directly interact with OSNs and influence targeting. In total, 23 cDNAs were found to be spatially varied in expression. As expected, all identified cDNAs were differentially expressed within the nascent EPL/GL, as confirmed by DAPI labeling and double-label *in situ *hybridization (see below). Expression was not necessarily exclusive to these layers, and was often observed in the mitral layer as well.

### Spatial differential expression of candidate cues at E17.5

**Figure 2:**
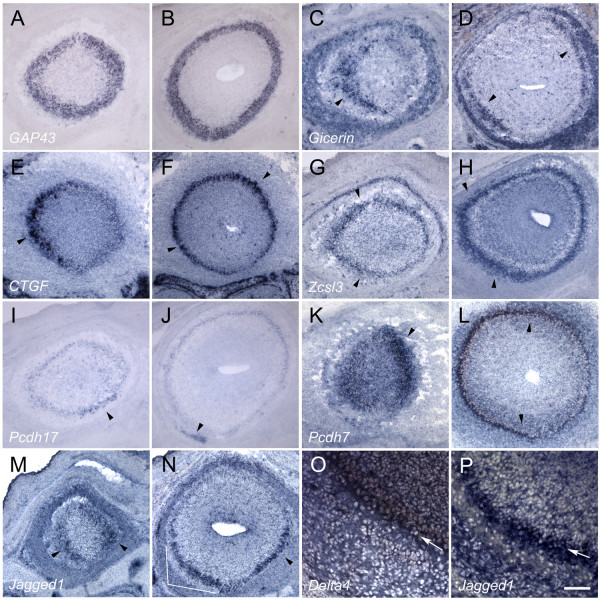
**Spatially differentially expressed genes in E17.5 bulb**. Bulb sections are shown in pairs. The first column corresponds to anterior bulb and the second column corresponds to middle bulb. Medial is to the right and dorsal is towards the top. A-B) *GAP43 *expression is uniform within the EPL and mitral layers. C-D) *Gicerin *expression is primarily lateral in anterior bulb (arrowhead), with weak expression medially. In middle bulb, expression of *Gicerin *is symmetric, with signal in both VL and DM bulb (arrowheads). Weak expression connects these two domains here and in the posterior bulb (data not shown). E-F) *Connective tissue growth factor (CTGF) *expression is similar to *Gicerin *anteriorly, with variable expression (arrowhead). In middle bulb, *CTGF *is symmetrically expressed (arrowheads) in the VL and DM domains, with weaker expression connecting the two domains. G-H) *Zinc finger csl-type containing 3 *(*Zcsl3*) is expressed in a gradient that extends from the dorsal to ventral, with highest expression along the VL aspect in anterior and middle bulb (arrowheads). I-J) *Protocadherin17 *expression is weakly present uniformly in the bulb, but regional higher expression can be observed in the VM aspect anteriorly (arrowhead). In the middle bulb, expression can be detected in the VL nerve layer (arrowhead). K-L) *Protocadherin7 *expression is strongest in the medial bulb anteriorly (arrowhead), but then moves towards the lateral surface in the middle bulb (arrowheads). Weaker expression is still present in the medial surface. M-N) *Jagged1 *expression in both anterior and middle bulb is symmetric about the ML axis (arrowheads). Note that this symmetry occurs only in the ventral bulb, unlike *Gicerin *and *CTGF*. O) *Delta4 *expression in the bulb is essentially restricted to the mitral layer (arrow). The section is counterstained with DAPI to show the location of cells in the EPL. P) Higher magnification view of *Jagged1 *expression shown in 2N. Section has been counterstained with DAPI to show the location of cells in the EPL. *Jagged1 *expression extends into the EPL (arrow); compare with 2O. Scale bar = 100 μm (2A-N); 25 μm (2O-P).

We used *GAP43 *expression [22] and DAPI staining as controls for our initial experiments. We found *GAP43 *to be evenly distributed throughout the mitral and EPL layers (Fig. 2A–B). DAPI staining showed that slightly more cells are observed laterally in the anterior bulb, but in the middle and posterior bulb the distribution of cells appears uniform (data not shown). These controls served to distinguish whether or not any of our observed *in situ *patterns simply reflected the presence of increased numbers of cells at a given location within the bulb.

We identified a diversity of patterns that are spatially differentially expressed within the nascent EPL/GL. We found that many of our positive cDNAs corresponded to genes that are either secreted or are transmembrane-bound proteins. *Gicerin *is an immunoglobulin superfamily member that is a homophilic cell-adhesion molecule [23]. *Gicerin *is expressed differentially in the lateral aspect of the anterior bulb (Fig. 2C). However, not all cells in this lateral domain express *Gicerin*, as the pattern appears stippled. Expression is also observed in the nerve layer. Strikingly, in the middle bulb, expression of *Gicerin *appears symmetric in some areas about the ML and DV axes, with high levels observed in the DM and VL bulb (Fig. 2D). In more posterior bulb, these expression domains gradually become connected with one another along the DL surface, forming a crescent-shaped expression domain (data not shown). Expression in the VM aspect, however, remains low throughout the AP extent of the bulb.

*Connective tissue growth factor*, or *CTGF *[24], is a secreted protein that is a member of a small family of genes which interact with the extracellular matrix and growth factors, and are important for angiogenesis [25]. In the anterior bulb, *CTGF *is localized to the lateral aspect (Fig. 2E). Like *Gicerin*, this expression is not uniform and significantly higher expression occurs within a subset of these cells. In the middle and posterior bulb, as with *Gicerin*, expression is symmetric as higher *CTGF *expression is detected along the DM and VL surfaces (Fig. 2F). *CTGF is *weaker in the DL aspect and noticeably reduced in the VM bulb. *CTGF *is also expressed in cartilage and connective tissue surrounding the bulb.

*Zinc finger csl-type containing 3 (Zcsl3) *is a protein of unknown function [26]. Throughout the AP axis, *Zcsl3 *is expressed in a gradient-like pattern that extends from the DM to the VL bulb (Fig. 2G), although expression tends to shift more ventrally in the middle bulb (Fig. 2H).

Protocadherins have been shown to possess both homophilic and heterophilic adhesion properties [27]. *Protocadherin 17 *(*Pcdh17*; [28]) is expressed throughout the bulb, but is enhanced in the anterior bulb along the VM aspect in a small subset of cells that extend into the EPL (Fig. 2I). In the middle bulb, a small subset of cells in the VL nerve layer expresses this gene (Fig. 2J). *Protocadherin 7 *(*Pcdh7*; [28]) is expressed anteriorly most strongly along the medial aspect, although some signal can be seen laterally (Fig. 2K). Just beyond the anterior bulb, this pattern is reversed and *Pcdh7 *becomes strongest laterally, although some expression can still be detected medially (Fig. 2L). This pattern is maintained throughout the posterior bulb (data not shown).

*Jagged1*, a ligand for *Notch*, has been shown to be required for proper endothelial branching during angiogenesis [29]. In the anterior bulb, *Jagged1 *is symmetrically expressed about the ML axis (Fig. 2M). Expression of *Jagged1 *in these domains appears more even than that of *Gicerin *or *CTGF*, and no obvious high or low-expressing cells are observed within the expression domain. In the middle bulb, *Jagged1 *maintains this ML symmetry, but weaker expression connects the two subdomains (Fig. 2N). A similar pattern is observed in posterior bulb (data not shown).

Although many of our genes are expressed in the nascent mitral layer, we emphasize that at least some expressing cells are located within the developing EPL. All of our *in situ *hybridization experiments were counterstained with DAPI so that we could correlate the location of the observed signal with the position of the nascent mitral and external plexiform layers. *Delta4*, another ligand for *Notch *[30], is expressed primarily within the mitral layer, and does not extend into the EPL (Fig. 2O). This can be seen both by DAPI staining and by the relatively compact expression pattern for this gene (Fig. 2O). *Jagged1*, on the other hand, extends beyond the mitral layer into the EPL, and its expression domain is broader than that of *Delta4 *(Fig. 2P). We further demonstrate that these genes are expressed in the nascent EPL/GL using double-label experiments and temporal analysis of their expression patterns, as described below.

These expression patterns underscore the strong spatial heterogeneity within the external plexiform layer. Moreover, they emphasize that there is variation along all three axes of the bulb. They also show for the first time that the bulb does indeed express genes that appear symmetric about the ML axis.

### A spatially differentially expressed putative microRNA

**Figure 3:**
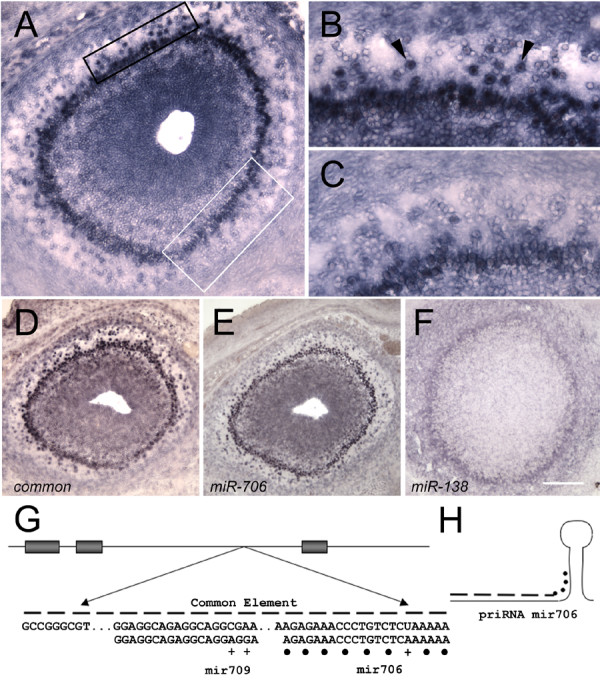
**Common element expression**. Medial is to the right and dorsal is towards the top. A) E17.5 coronal bulb showing the typical punctate pattern observed with any one of 17 cDNA probes containing the common element. A) Signal is observed in the nascent EPL/GL, mitral, and granule layers. Weaker expression is observed in both the nascent EPL/GL and mitral layers along the VM surface (white box) as compared to the rest of the bulb (e.g. black box). B) Higher magnification of the black boxed area shown in (A). Note strong nuclear expression in nascent EPL/GL (arrowheads). C) Higher magnification of the white boxed area shown in (A). Note fewer cells and weaker staining as compared with (B). This is not due to a reduced number of cells in this area, as a significant number of nascent EPL/GL cells can be seen to be present that are unstained. D) Expression pattern using the 134 bp common element as probe. E) Expression pattern using a dig-labeled LNA-oligo corresponding to *miR-706*. (D) and (E) are qualitatively similar to (A). F) Expression pattern using an LNA oligo for *miR-138*. The pattern is distinct from *miR-706*, with no punctate expression. Scale bar = 100 μm for A; 50 μm for C, D; 75 μm for D, E, F. G) The common element is contained within an intron or 3' untranslated region. Only a portion of the 134 bp conserved sequence is shown. Sequence contained within the element matches that of *miR-709 *at 17/19 positions (mismatches indicated by "+"). For *miR-706*, a subset of the cDNAs matched the sequence perfectly, with others matching at 21/22 or 20/22 positions. The most commonly observed mismatched nucleotide occurred at the 3' end (indicated by a "+"). H) The remainder of the element corresponds to sequence upstream of the presumed pri-miRNA (shown as dashed lines and filled circles) of *miR-706*.

Among the remaining 17 cDNAs, significantly enhanced expression was observed along the DV axis. The patterns observed among all of these positives were remarkably similar, and one representative example is shown in Figure 3. Within the nascent EPL/GL, these genes are expressed in a punctate pattern (Figs. 3A–B). This punctate expression occurs throughout the nascent EPL/GL, but significantly fewer cells were labeled in the VM aspect (compare Figs. 3A–B with Figs. 3A,3C).

Bioinformatic analysis showed that all 17 cDNAs contained intronic or 3' untranslated sequence (Fig. 3G). Pair-wise alignment identified a 134 basepair common element that contained two microRNA (miRNA) sequences, *miR-706 *and *miR-709 *(Fig. 3G). The remainder of the 134 bp element was ~ 95% identical to sequence immediately upstream of the *miR-706 *sequence [31], corresponding to what we assume is part of the primary miRNA (pri-miRNA) sequence for *miR-706 *(Fig. 3H).

The expression pattern we observed may therefore reflect the expression of *miR-706 *pri-miRNA. We obtained locked nucleic acid (LNA)-modified oligos corresponding to *miR-706 *and *miR-138 *(a control miRNA known to be expressed in the bulb; [32]). We performed *in situ *hybridization with the LNA oligos as well as with the 134 basepair common element. Hybridization patterns using the common element were identical to that observed with our 17 cDNAs (compare Fig. 3A with 3D). Although the signal for the *miR-706 *probe was weaker than that of the common element, punctate signal was also observed in the nascent EPL/GL in a similar pattern as well (compare Fig. 3A with 3E). These results contrast with those for the *miR-138 *probe, which bound weakly and uniformly to the nerve layer and EPL (Fig. 3F). The miRNA *in situ *patterns that have been published in the mouse generally show uniform expression within a given layer or structure (e.g. *miR-138*), and/or are detected in specific cell-types. The pattern of the *miR-706 *probe suggests, however, that miRNAs may also be present selectively within subsets of cells in a spatially distinct fashion. Additional studies are required to determine the possible targets of this putative miRNA.

### Three-dimensional reconstruction of gene expression

Our identified expression patterns show that the bulb can be subdivided into different domains that are distinguished molecularly by the expression of these candidate genes. These domains have not been previously described, as only a handful of genes are known to be differentially expressed within a given layer of the bulb [33-35]. We were surprised by the complexity of the observed patterns, as prior mapping studies had suggested the existence of DV cues in the bulb [12], and theoretical models had suggested the existence of symmetric cues to label medial and lateral bulb [36]. Our observed expression patterns, however, do not readily fit any of these prior suggested models. For example, *Gicerin *symmetrically labels the DM and VL bulb, but symmetry about these axes has not been previously predicted. To obtain a more detailed understanding of the expression domains distinguished by these individual genes, we generated a three-dimensional reconstruction of gene expression for these genes within the bulb.

**Figure 4:**
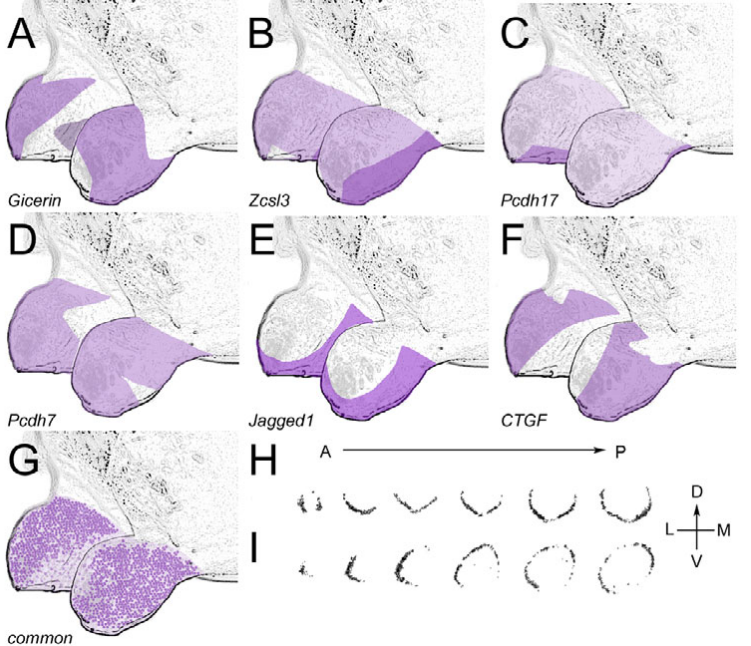
**Three-dimensional reconstruction of gene expression at E17.5**. A) *Gicerin*. B) *Zinc csl-type containing 3*. C) *Protocadherin17*. D) *Protocadherin7*. E) *Jagged1*. F) *Connective tissue growth factor*. G) Putative microRNA-containing common element. H) Examples of *in situ *hybridization data used to create the *Jagged1 *reconstruction (Fig. 4E). Six coronal sections are shown taken from anterior to posterior bulb. Dorsal is up and medial is to the right. I) Examples of *in situ *hybridization data used to create the *Connective tissue growth factor *reconstruction (Fig. 4F).

To produce these recreations, coronal sections from E17.5 bulbs were obtained every 60–80 μm along the AP extent of the bulb for every gene. The images of the hybridized sections were combined using ImageJ [see Methods]. The projection of the stacked images was then superimposed upon a cartoon representation of the olfactory bulb for ease of visualization. A subset of the images used to create the *Jagged1 *and *CTGF *stacks are shown in Figure 4H–I, and the corresponding reconstructions are shown in Figure 4E–F. Reconstructions of the other genes are also shown in Figure 4. These reconstructions show that domains of the bulb along the AP, DV, and ML axes can be uniquely specified by the combinatorial expression of these genes. These data support a model where molecular heterogeneity in the nascent EPL/GL would provide spatial guidance information to OSN axons as they converge within the olfactory bulb.

### Temporally dynamic expression of candidate cues during development – E14

The first OSNs are known to extend an axon towards the developing bulb at E11–12, and "wait" at the periphery as the bulb develops [37]. *Sema3A *expression at the periphery of the bulb, expressed by ensheathing glia, plays a role in maintaining OSNs outside of the bulb [38]. It is known that mitral cell reorientation requires innervation by a small population of early OSNs [21] that enter the bulb in a medial-to-lateral progression [39]. Why this innervation proceeds initially along the medial surface is not known. One possibility would be that additional cues, in conjunction with *Sema3A *expression, serve to guide OSN axons to their position within the bulb. We therefore examined the expression of our candidate cues at E14 for differential expression.

**Figure 5:**
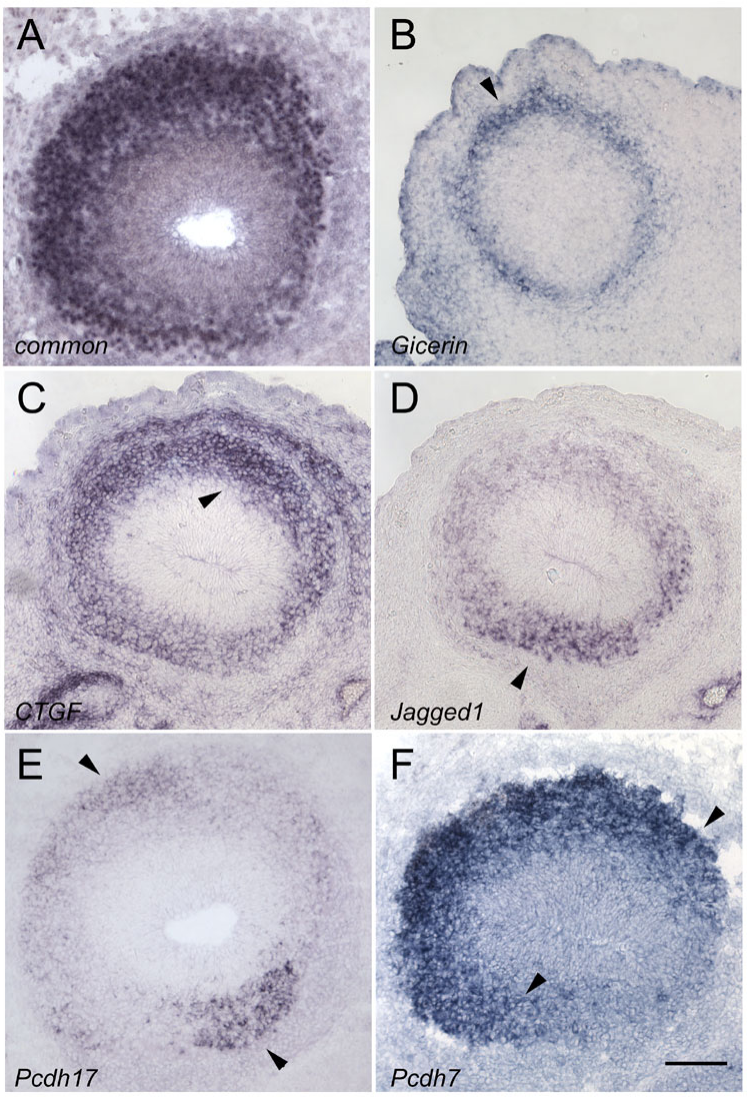
**E14 spatial differential expression**. Medial is to the right and dorsal is towards the top. A) The putative miRNA is expressed strongly in the DL bulb and weakly in the VM aspect. B) *Gicerin *expression at E14 is elevated in the DL bulb (arrowhead). C) *Connective tissue growth factor *expression is higher in the DM bulb (arrowhead). D) *Jagged1 *expression is elevated ventrally (arrowhead). E) *Protocadherin17 *is expressed in two distinct layers in the VM mantle (arrowhead). Expression can also be detected in the DL aspect (arrowhead). F) *Protocadherin7 *is expressed broadly within the bulb in a pattern that roughly complements that of *protocadherin17 *(arrowheads), with very low expression observed ventromedially. Scale bar = 100 μm.

All of our identified genes are differentially expressed at E14 in the superficial mantle layer. The putative miRNA containing sequence, for example, is expressed strongly in the DL bulb, with weak expression in the VM aspect (Fig. 5A). *Gicerin *is expressed more highly in the DL bulb as well, with weak expression in VL bulb (Fig. 5B). *CTGF*, on the other hand, is expressed more highly in the DM bulb (Fig. 5C). *Jagged1 *is expressed more strongly in the ventral aspect (Fig. 5D). *Pcdh17 *and *Pcdh7 *label complementary domains (Fig. 5E–F). *Pcdh17 *strongly labels two populations of cells in the VM bulb, with lighter, more uniform staining observed internally, and stronger, more selective staining externally. Faint staining is also observed in the DL bulb, symmetrically opposing the VM expression. *Pcdh7*, in contrast, strongly labels a large number of cells throughout the bulb except those along the VM surface.

Several of the observed patterns at E14 mimic those seen at E17.5. For example, the putative miRNA is weakly expressed in the VM bulb at both stages. Similarly, the dual-layer expression of *Pcdh17 *in the VM bulb at E14 is reflected at E17.5. *Jagged1 *is ventrally expressed at both stages. On the other hand, other genes change in expression between E14 and E17.5. *Gicerin*, for example, is expressed most strongly at E14 in DL bulb, but at E17.5 is present more strongly in the DM and VL bulb. *CTGF *also shifts in expression along different axes from E14 to E17.5, increasing in expression laterally and anteriorly as development proceeds.

### Temporally dynamic expression of candidate cues during development – P3

We next examined the expression of these various candidate cues at postnatal stages. While convergence has been shown to occur at E17.5 for OSNs expressing the P2 odorant receptor, other OSN populations do not converge until later stages, up to postnatal day 3 [40,41]. The existence of relatively late converging OSNs prompted us to examine the expression of these genes at P3.

**Figure 6:**
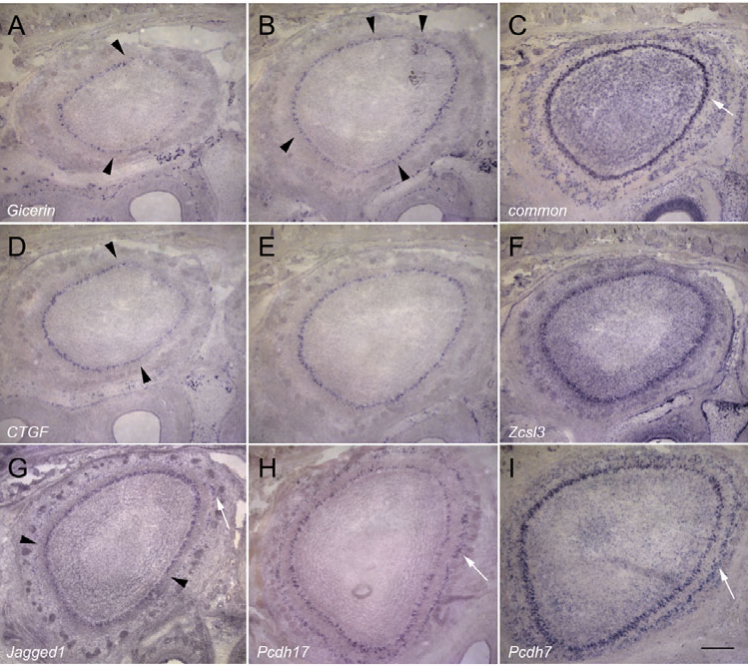
**P3 spatial differential expression**. Medial is to the right and dorsal is towards the top. A) *Gicerin *expression in the anterior bulb is enhanced in the lateral aspect (arrowheads). B) *Gicerin *expression in the middle bulb displays symmetric expression, with increased expression along the DM and VL aspects. Weaker expression connects the two domains (arrowheads). C) The putative miRNA-containing common element appears uniformly distributed in the mitral, external plexiform, and glomerular layers. Arrow indicates EPL expression. D) *Connective tissue growth factor (CTGF) *expression in the anterior bulb is predominantly lateral (arrowheads). E) *CTGF *expression in the middle bulb is uniformly distributed. F) *Zinc finger csl-type containing 3 (Zcsl3) *expression at P3 is uniform in all aspects of the bulb. G) *Jagged1 *expression is differentially expressed in the dorsal aspect of the bulb (region between arrowheads). Expression can also be observed more peripherally deep to the GL (arrow). H) *Protocadherin17 *expression is uniform in all aspects. Expression is detected in the mitral and glomerular (arrow) layers. I) *Protocadherin7 *expression is uniform in all aspects of the bulb. Similar to *protocadherin17*, expression is observed in the mitral and glomerular (arrow) layers. Scale bar = 200 μm.

At P3, *Gicerin *is expressed in the anterior bulb more highly in the lateral aspect (Fig. 6A). In the middle bulb, expression can be seen in a complementary, symmetric pattern (Fig. 6B). *CTGF *is also expressed differentially in the lateral aspect of the anterior bulb (Fig. 6D), but in the middle and posterior bulb, this expression becomes more uniform (Fig. 6E). In contrast to *Gicerin *and *CTGF*, the putative miRNA is now expressed uniformly within the EPL (Fig. 6C). Similarly, *Zcsl3 *(Fig. 6F), *Pcdh17 *(Fig. 6H) and *Pcdh7 *(Fig. 6I), are uniformly expressed. However, *Jagged1 *is still differentially expressed at P3. But unlike it's expression at E14 and E17.5, higher expression is observed along the dorsal aspect (Fig. 6G).

As with the data regarding expression at E14, these patterns indicate the temporally dynamic nature of expression by these various genes. *Gicerin*, for example, is expressed most highly in the DL bulb at E14, but is DM and VL in both the E17.5 and P3 bulb. *Pcdh17 *is VM and DL at E14, VM at E17, and uniform at P3. We note that, consistent with our initial observations at E17.5, *Zcsl3*, the putative miRNA, *Jagged1, Pcdh17*, and *Pcdh7 *expression can clearly be detected in the EPL, GL, and mitral layers.

### Lack of precise colocalization with *Tbr1*

We have hypothesized that cells within the EPL express guidance cues for OSNs. Genetic studies using the *Tbr1 *mutant mouse resulted in the ablation of most projection neurons in the bulb [7]. Projection neurons include mitral cells as well as internal and middle tufted cells [1]. As targeting is unaffected in *Tbr1 *mutant mice, a major problem has been to determine what cell-types within the bulb remained that could be important for this process. If our genes are expressed in cells that provide guidance information to OSNs, we would predict that at least some cells that express our candidate genes do not express *Tbr1*, and would not be ablated in the mutant.

**Figure 7:**
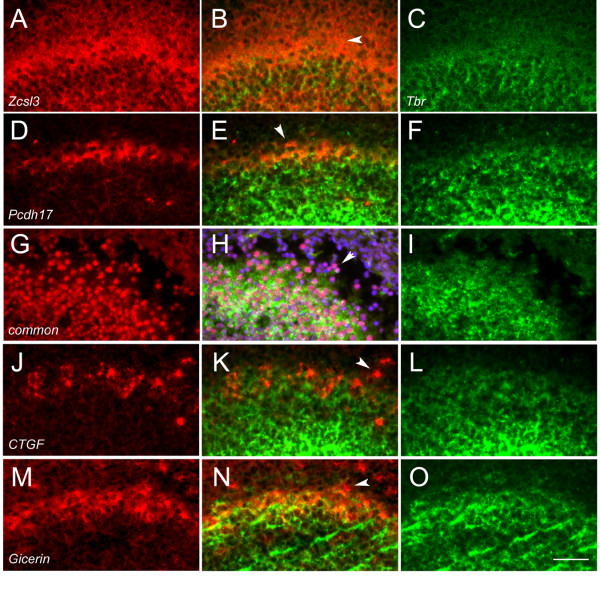
Double-label *in situ *hybridization with *Tbr1*. Nascent EPL/GL is dorsal and the granule layer is ventral in each section. Arrowheads in all panels indicate cells that express candidate genes but do not express *Tbr1*. A-C) *Zinc finger csl-type containing 3 (Zcsl3) *(red)/*T-box brain gene 1 (Tbr1) *(green). Note *Zcsl3 *expression extends beyond the *Tbr1 *expression domain. D-F) *Protocadherin17 *(red)/*Tbr1 *(green). G-I) Typical punctate pattern observed using a cDNA probe containing the common element (red)/*Tbr1 *(green)/DAPI (blue). J-L) *Connective tissue growth factor *(red)/*Tbr1 *(green). M-O) *Gicerin *(red)/*Tbr1 *(green). Scale bar = 100 μm.

Prior studies have shown that *Tbr1 *is expressed in cells distributed from the ventricle to the nascent mitral/tufted layer at E16.5 [42]. We found a similar distribution of *Tbr1*-positive cells at E17.5. We performed double-label experiments with *Tbr1 *and with our genes to determine what overlap exists in expression. *Zcsl3 *is enhanced in expression laterally (Fig. 7A). In contrast, *Tbr1 *expression (green) in this same section is widespread in the granule and mitral/tufted layer (Fig. 7C), as previously described [42]. Merging the two images shows that expression of *Zcsl3 *occurs in cells that extend into the nascent EPL/GL which do not express *Tbr1 *(Fig. 7B). Similarly, *Pcdh17 *is enhanced in VM cells in the anterior bulb (Fig. 7D). A subset of these cells do not overlap in expression with *Tbr1 *(Fig. 7E,7F). The putative miRNA identified in our screen is also expressed in nascent EPL/GL cells that do not express *Tbr1 *(Fig. 7G–I). Finally, a subset of *CTGF *(Fig. 7J–L) and *Gicerin *(Fig. 7M–O) expression also occurs within cells that do not express *Tbr1*. Thus, while overlap with *Tbr1*-expressing cells does occur, *Tbr1*-negative cells that express our genes are present within the bulb. Perhaps most interestingly, these *Tbr1-*negative cells are primarily located in the regions where the differential expression of our genes appears strongest. For example, *Zcsl3 *is spatially differentially expressed in the lateral bulb, and many of these cells have no detectable *Tbr1 *expression (Fig. 7B).

### Expression of candidate cues are affected in chemically ablated mice

While we have shown the existence of a number of genes demarcating different domains within the bulb, it remains unknown whether or not they are functionally relevant for OSN guidance. Unfortunately, no *in vitro *system exists in the olfactory system to test this hypothesis, and genetic analysis has remained the primary means for assessing function. To more rapidly determine the potential role of these genes in targeting, we decided to employ a chemical ablation approach.

The olfactory epithelium is continuously being regenerated during adult life. Prior studies have shown that *Tyrosine hydroxylase (TH) *expression is drastically reduced following intranasal ablation with zinc sulfate [43]. As regeneration proceeds, *TH *expression gradually recovers. Although *TH *is an intracellular enzyme, it has been proposed that this recovery is dependent upon afferent input from regenerating OSNs. This process, although poorly understood, is thought to be a result of transneuronal regulation of glomerular neurons by OSNs [43].

**Figure 8:**
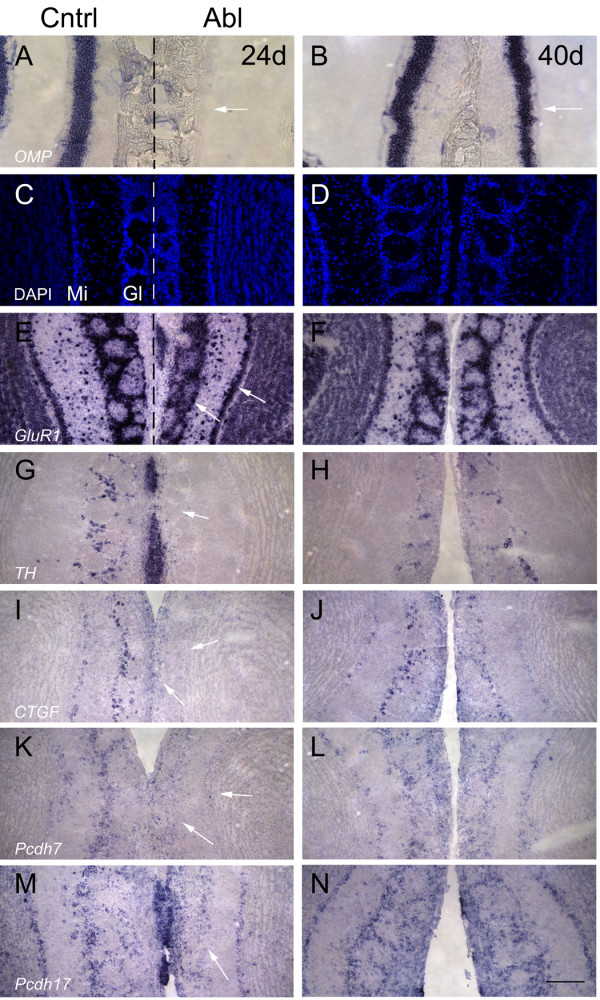
**Expression after chemical ablation**. In all panels, the left half corresponds to the unablated side and the right half corresponds to the ablated side. Panels A, C, E, G, I, K and M correspond to images taken from mice that were sacrificed 24 days post-ablation. Panels B, D, F, H, J, L and N correspond to images taken from mice that were sacrificed 40 days post-ablation. A) *Olfactory marker protein (OMP) *expression in the epithelium of 24 day ablated mice. No expression of *OMP *is observed in the epithelium on the ablated side (compare right half (arrow) with left half). B) *OMP *expression in the epithelium of 40 day ablated mice. Recovery of *OMP *expression has begun on the ablated side (right side), although this expression pattern is thinner and less robust than that of control (arrow). C) DAPI staining of bulbs from 24 day ablated mice shows significant reduction in glomerular size, but no apparent decrease in cell number. D) DAPI staining of bulbs from 40 day ablated mice show no dramatic differences compared to control, although glomerular size still appears somewhat reduced. E) *Glutamate receptor subunit 1 (GluR1) *expression in bulbs from 24 day ablated mice. Note stronger glomerular expression of *GluR1 *on the unablated side relative to the ablated side. However, *GluR1 *expression in the mitral layer was slightly but visibly stronger on the ablated side relative to control (arrows). F) *GluR1 *expression has returned in 40 day ablated animals to levels similar to control. G) *Tyrosine hydroxylase (TH) *expression in 24 day ablated animals is dramatically reduced (arrow). H) *TH *expression in 40 day ablated animals. Expression of *TH *has returned to levels similar to that of control. I) *Connective tissue growth factor (CTGF) *expression in 24 day ablated animals. On the unablated side, *CTGF *is expressed in both the mitral/tufted and glomerular layer. No expression in either layer is observed in the ablated animal (arrows). J) *CTGF *expression in 40 day ablated mice. Expression levels are slightly weaker than on the control side but is still clearly visible in mitral and glomerular layers. K) *Protocadherin7 (Pcdh7) *expression, like *CTGF*, is also detected in the mitral and glomerular layers in adult, although expression in the mitral layer is weak and inconsistent. On the ablated side, expression is reduced in both layers, but weak expression is still visible in some cells (arrows). L) *Pcdh7 *expression in 40 day ablated mice. Observed expression of *Pcdh7 *approximates that of the control bulb. M) *Pcdh17 *expression in 24 day ablated mice. Expression is present in both the mitral and glomerular layers. However, on the ablated side, only expression in the glomerular layer is affected (arrow). N) *Pcdh17 *expression in 40 day ablated mice. Expression has recovered and approximates that of control. Scale bar = 125 μm (7A-D); 200 μm (7E-N).

We decided to ask whether or not this ablation paradigm could be used to determine whether our candidate genes are expressed in cells that interact with OSNs. We would expect that if they were, then their expression, like that of *TH*, would change in response to OSN deafferentation. We ablated OSNs in one nares of eight-week old mice with a solution of zinc sulfate, leaving the other nares alone. We determined the success of the ablation based on expression of *OMP *in the epithelium. As shown in the right half of Fig. 8A, at 24 days post-ablation, essentially no *OMP *is observed in the ablated epithelium. Small, isolated pockets of *OMP *expression were occasionally detected in some ablated epithelia, presumably due to a lack of access to these regions by the zinc sulfate solution (data not shown). By 40 days post-ablation, *OMP *expression has started to recover, although this expression is still somewhat thinner when compared to the control side (compare right and left halves of Fig. 8B). We did not examine later timepoints. We found that the rate of *OMP *recovery was somewhat variable, with some epithelia showing OMP expression by 30 days, while others showed little or no expression. We therefore based our experiments in the bulb on the pattern of *OMP *expression in the epithelium, and not by the number of days post-ablation.

We found the bulb on the ablated side is smaller as previously reported [43] due to a significant reduction in the size of the nerve and glomerular layers (data not shown). Based on DAPI staining, the number of cells in the glomerular layer did not appear to be reduced (Fig. 8C–D), consistent with previous studies showing that little or no cell death results from the ablation process [43,44]. We found that *GluR1 *expression appeared to be reduced in the glomerular layer and increased in the mitral layer at 24 days (Fig. 8E) but was equal in expression between ablated and control bulbs by 40 days (Fig. 8F). This is reminiscent of alterations in *GluR1 *expression in naris occluded animals [45]. We confirmed that *TH *was absent from the ablated side at 24 days (Fig. 8G) but recovered by 40 days post-ablation (Fig. 8H).

We then tested a subset of our genes on these ablated animals. Interestingly, we found a variety of different responses to the ablation. *CTGF *is expressed in the adult in the glomerular and mitral layers (Fig. 8I). This expression is absent in bulbs on the ablated side at 24 days (Fig. 8I) but recovers by 40 days (Fig. 8J). *Pcdh7 *is expressed in the glomerular layer in adults, with weaker, more scattered expression in the mitral layer (Fig. 8K). Like *CTGF*, both layers are affected in the ablated side at 24 days (Fig. 8K), but expression recovers by 40 days post-ablation (Fig. 8L). *Pcdh17 *is also expressed in the glomerular and mitral layers (Fig. 8M). However, in the ablated side, only the glomerular layer expression of *Pcdh17 *is affected, while mitral layer expression remains constant (Fig. 8M). Expression in the glomerular layer returns to control levels by 40 days (Fig. 8N). We did not find any differences in expression for the putative miRNA or *Zcsl3 *in bulbs in ablated animals (data not shown). Based on prior studies showing *TH *regulation by OSN innervation, we conclude that several of our genes are likely to be expressed in cells that interact directly with OSNs.

### *Pcdh7 *and *Pcdh17 *are expressed in subsets of OSNs

Two of our genes, *Pcdh7 *and *Pcdh17*, are members of the cadherin superfamily. Protocadherins are known to possess weak homophilic adhesion properties *in vitro *[46,47]. In one model, protocadherins expressed by OSNs would bind their cognate partner expressed by nascent EPL/GL cells within the bulb. The specificity of these interactions would contribute towards the final glomerular convergence pattern.

**Figure 9:**
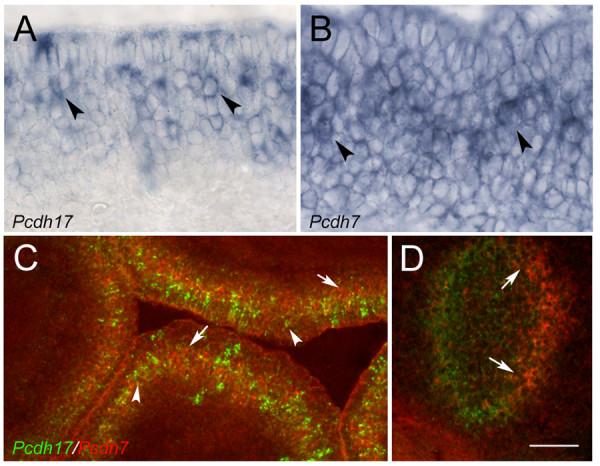
Expression of *protocadherin17 *and *7*in the epithelium. A) *Protocadherin17 *(*Pcdh17) *expression in the OE is localized to subsets of cells in the neuronal layer (black arrowheads). Expression is also seen in occasional cells in the sustentacular and basal layers. B) *Protocadherin7 *(*Pcdh7*) expression in the epithelium is localized to subsets of cells in the neuronal layer (black arrowheads). C) Double-label *in situ *hybridization using *Pcdh17 *(green) and *Pcdh7 *(red) as probes. Although overlap in expression is occasionally observed, most cells express either *Pcdh17 *(white arrowheads) or *Pcdh7 *(arrows). D) Double-label *in situ *hybridization experiment showing domains of expression of *Pcdh17 *(green) and *Pcdh7 *(red) in the anterior bulb. The localized expression of *Pcdh17 *at the VM surface partially overlaps with that of *Pcdh7 *(domain between arrows) in anterior bulb. Scale bar =25 μm (A,B), 100 μm (C,D).

We therefore examined *Pcdh7 *and *Pcdh17 *expression in the epithelium. Interestingly, we found both genes are expressed in subsets of OSNs (Figs. 9A–B). Double-label fluorescent *in situ *hybridization shows that, although some cells express both *Pcdh17 *and *Pcdh7*, others express one or the other protocadherin (Fig. 9C). Within the bulb, fluorescent double-label *in situ *experiments show a similar phenotype, as *Pcdh7 *expression overlaps with that of *Pcdh17*, but distinct domains of the bulb are uniquely labelled with one or the other protocadherin (Fig. 9D). The expression of these cadherins in both the epithelium and in the bulb is consistent with a model in which these molecules contribute towards the establishment of the topographic map.

## Discussion

To our knowledge, no cell-type or gene expressed within the bulb has been functionally proven to provide spatial guidance information to OSNs. *Sema3A*, for example, has been genetically shown to affect OSN targeting, but this is due to its expression in ensheathing glia [38] which migrate to the nerve layer from the epithelium. A number of genes are uniformly expressed within cells derived from the bulb (e.g. *Sema3A *[48]), but it is difficult to understand how, given this uniform expression, these genes would guide OSN axons to different domains within the bulb. The lack of viable potential guidance cues has led instead to genetic approaches that ablated either whole classes of cell-types within the bulb (e.g. *Tbr1*; [7]) or eliminated much of the bulb entirely (e.g. *Extratoes*; [11]). The absence of suitable molecular candidates that could potentially guide OSN axons to subdomains of the bulb has strongly inhibited any study of the role of the bulb in guidance.

In contrast, studies manipulating odorant receptor expression have led to models where the odorant receptor plays a significant role in guidance [3,4,49]. The recent discovery that the *Kirrel 2/3 *and *Ephrin/Eph A5 *expression are controlled by odorant receptor activity provides new insight into how receptor choice influences glomerular positioning. However, in all of these receptor swap experiments, it is clear that the receptor alone does not mediate all aspects of glomerular targeting. The dorsal-ventral position of the OSN within the epithelium is thought to provide position-dependent information as well [9]. But what genes comprise these position-dependent determinants, and what potential partners are bound by such determinants are still unknown. Moreover, why OSNs innervate both a medial and a lateral glomerulus has not been explained by studies manipulating odorant receptor expression. The presence of gradients [36] or of mirror-symmetric expression patterns within the bulb [50] have been suggested as potential explanations for how topographic OSN projection patterns are established. However, considerable disagreement exists over the role of the bulb in guidance [9].

The role of bulbar projection neurons in guiding OSN axons has been examined using different genetic paradigms [7,8]. These studies have suggested that these cells are not necessary for topographic convergence of OSNs. While these analyses only examined OSNs that express the P2 odorant receptor, mitral cell tracing studies also do not necessarily support a role for these cells in guidance. Mitral cell dendritic arbors initially are distributed among several glomeruli, but are refined during the first week of postnatal development to a single glomerulus [15]. It is difficult to understand, if mitral cells do indeed express the primary cues needed for OSN guidance, why such cues would be distributed among multiple glomeruli initially, and the dendritic arbor refined only after convergence has been completed. But despite the fact that these studies indicate that mitral cells may not play a primary role in guidance, genetic ablation studies removing the entire bulb [11] have shown that topographically correct convergence by P2-expressing OSNs does not occur. One interpretion of these results is that other cell-types within the bulb are important for providing guidance information to OSNs. We theorized that these cells correspond to those in the EPL and GL.

In this hypothesis-driven microarray-based experiment, we show that the nascent EPL/GL of the bulb can be subdivided into multiple domains based on the expression of our identified genes. At E17.5, the first time that convergence is observed [20], the anterior bulb can be distinguished molecularly both laterally (*CTGF*, *Gicerin, Jagged1*) and medially (*Jagged1*, *Pcdh7*). In the middle and posterior bulb, *Zcsl3 *is expressed in a gradient that extends from dorsal to ventrolateral, while a probe containing the *miR-706 *sequence is expressed in a subset of cells that span a similar region. Perhaps most interestingly, we find that *Gicerin*, *CTGF*, and *Jagged1 *are expressed in a symmetric manner. While it is well-known that OSNs project to medial and lateral glomeruli, the existence of mirror-symmetric cues has, to date, only been hypothesized [50]. However, our patterns suggest that this medial-lateral symmetry is more complex than previously thought. *Gicerin and CTGF *are roughly symmetric about both the DV and ML axes, while *Jagged1 *appears symmetric only around the ML axis. Thus, each hemi-bulb can be further distinguished based on dorsal or ventral expression of *Gicerin *and *CTGF*.

Several lines of evidence are consistent with the expression of these candidate cues within cells of the EPL. First, we show by counterstaining with DAPI that expression of these genes does not colocalize precisely with the nascent mitral layer. Second, we show at P3 and in adult that many of these genes are expressed in the EPL and GL, consistent with their expression at E17.5 in the nascent EPL/GL. Finally, we show that there are populations of cells at E17.5 that express our candidate genes but do not express *Tbr1*, a marker for projection neurons. Thus, we have shown using a variety of approaches that, in addition to mitral layer expression, nascent EPL/GL cells also express these genes.

Interestingly, in addition to the fact that not all cells that express our genes express *Tbr1*, the cells that do not express *Tbr1 *are those that show most clearly the differential expression patterns observed with our genes. For example, *Pcdh17 *is strongly and selectively expressed in a subset of cells in the VM aspect of the anterior bulb. These same cells do not express *Tbr1*. Thus, in *Tbr1 *mutant animals, we predict that these cells would not only be present, but that the observed differential expression patterns would also still be present. These cells may therefore provide a potential solution to the problem of which cells within the bulb could express guidance cues important for OSN guidance.

Mitral cells are known to mature in a medial-to-lateral fashion from E14–E17 [39], and a rostral-caudal wave of development occurs during glomerular formation [21]. However, these developmental events cannot readily explain our various observed patterns. For example, *Gicerin *and *CTGF *are expressed in subsets of cells in the lateral domain in anterior bulb, while *Jagged1 *and *Pcdh7 *are expressed medially. Several of our genes are both medially and laterally expressed. It seems unlikely that these patterns can be explained solely on the basis of neuronal maturation events during development.

Are any of our identified cDNAs involved in guiding OSNs to the bulb? An alternative interpretation would be that these genes may be important for establishing intrabulbar circuits. External tufted cells are known to indirectly connect glomeruli innervated by a common population of OSNs [19]. Thus, these genes may act to establish these connections. Yet another potential function of these genes can be suggested based on their expression in the mitral layer. These genes may act to mediate connectivity between projection neurons and higher cortical synaptic partners. Finally, these genes may not be involved in axon guidance *per se*, but instead are important for events surrounding synaptogenesis.

To provide support for the hypothesis these genes are important for guiding OSN axons, we used a chemical ablation approach [43]. Expression of *Tyrosine hydroxylase *(*TH*) is known to be down-regulated initially in ablated animals, but reappears as reinnervation of the bulb proceeds. Thus, *TH *expression is influenced by OSN innervation. Similarly, this approach has also been used to study *Sema3A *expression in axotomized bulbs [51]. In these experiments, normal expression of *Sema3A *is greatly reduced in axotomized bulbs. Expression is instead upregulated near the site of axotomy. Two months after ablation, *Sema3A *expression reappeared at its normal location. These results indicate that cells which interact with OSNs can display molecular alterations in their expression profile upon deafferentation. We showed that *CTGF, Pcdh17*, and *Pcdh7 *were altered in expression in response to ablation. Based on prior studies of *TH*, we conclude that these genes are expressed in cells that interact with OSNs.

We note that several of our candidate cues correspond to genes known to be important for guiding axons elsewhere in the nervous system (*Pcdh7*, *Pcdh17*, *Gicerin*), or are involved in angiogenesis (*Jagged1*, *CTGF*). *CTGF *and *Gicerin *are known to mediate cell-cell adhesion *in vitro*. Cadherins have been shown to be play multiple roles during development, from neurite outgrowth to synaptogenesis [52]. The changing patterns of expression of *Pcdh7 *and *Pcdh17 *during development may signal multiple roles as OSN innervation of the bulb proceeds. *Pcdh7 *and *Pcdh17 *may operate initially as guidance cues based on their differential expression within the bulb and epithelium. After growth of OSNs into the bulb, they may then act secondarily in synaptogenesis. While definitive proof requires genetic manipulation of the individual candidates, these genes provide novel targets that will enable new approaches towards determining the role of the bulb in guidance.

## Conclusion

We have identified several genes whose expression patterns define various subdomains within the developing mouse olfactory bulb. We show that these genes are expressed in cells within the nascent EPL/GL and ML, and that some of these cells do not express *Tbr1*. Our regeneration paradigm suggests that these genes are expressed in cells that interact with OSNs. These genes therefore represent novel candidate guidance cues that may guide OSN axons to appropriate locations within the bulb.

## Methods

### Animals

Swiss-Webster mice were used for all experiments. The day a vaginal plug was observed was day P0.5. All protocols were approved by the Cornell IACUC.

### Laser microdissection and RNA isolation

E17.5 embryos were embedded in 3% agarose and fresh frozen in isopentane/liquid nitrogen as described [53]. LMD samples were collected into 30 μl of lysis buffer and RNA isolated using Gentra's PureScript kit. Total RNA yield was ~ 200 pg/LMD isolate using the Agilent PicoChip Bioanalyzer assay.

### RNA amplification

A modified T7 RNA polymerase amplification protocol was performed using an optimized T7 primer [34]. First-round aRNA was tailed with poly(A) polymerase, providing a template for T7-d(T) primer and the generation of a second round of aRNA. For the arrays, 20% of this aRNA was amplified a third time, and a portion labeled by priming with amine-terminal random hexamers and incorporating a modified amino-allyl dUTP [54]. Hybridizations were performed in 50% formamide, 3xSSC, 20 mM Tris 8.5, 0.1% SDS, 0.1 μg/μl salmon sperm DNA, and 0.1% BSA at 45°C using a Tecan HS400 machine and scanned on an Axon 4000B scanner.

### Microarray generation

PCR products from the NIA 15 k [55], 7.4 k [56], and 11 k BMAP (brainEST.eng.uiowa.edu) collections were resuspended in 1xSSC/0.005% sarkosyl and printed on Corning UltraGAPS slides using a custom-built microarrayer and Telechem 946 pins.

*Experimental design*. For each section, the four LMD samples were hybridized directly against one another using a loop design [see Additional File 1]. The directions of the loops (clockwise or counter-clockwise) were alternated along the AP axis to control for dye bias.

### Statistical analysis

Background measurements were not subtracted to reduce variation in weakly expressing genes [57]. Within slide normalization (print-tip loess) was applied [58] and a fixed effects linear model was employed to identify spatial differentially expressed genes [59]. For a typical gene *g*, the intensity value corresponding to the four different sub-regions of the bulb is denoted by *DM*_*k*_,*DL*_*k*_,*VM*_*k*_,*VL*_*k*_, where k represents the different sections taken along the AP-axis. The log transformation of these values is defined as *dm*_*k *_= log_2 _*DM*_*k*_, *dl*_*k *_= log_2 _*DL*_*k*_, *vm*_*k *_= log_2 _*VM*_*k *_and *vl*_*k *_= log_2 _*VL*_*k*_. To estimate the spatial gene expression for gene *g*, all of the spatial sections along the AP-axis are treated as replicates and used to fit four separate linear models for each of the comparisons of interest α_1 _= *dm*-*dl*, α_2 _= *dm*-*vm*, α_3 _= *vl*-*dl *and α_4 _= *vm*-*vl *. The linear model is written as

*M*_*ik *_= α_*i *_+ ε_*ik*_

where *i *= 1,,4 representing the four main comparisons of interest; *M*_*ik*_is a vector of log-ratios and var(ε_*ik*_) = σ_*i *_This model is equivalent to performing four separate series of one-sample comparisons on four separate sets of data. Estimates of the coefficient of variation, moderated *t*-statistics, and adjusted p-values were calculated using *lmFit *in the library *limma *[59]. Comparison of this model with other analysis options, as well as gene lists for the individual groups, are presented as an additional file [see Additional File 1].

### *In situ *hybridization

Probes were generated from the original clone printed on the array. Hybridization to sections was essentially as described [34]. Fixed sections were treated with 10 μg/ml proteinase K prior to hybridization at 60°C. Double label *in situ *hybridization was performed with digoxigenin and biotin-labeled probes and detected using the Fast Red/HNPP substrate or the TSA Renaissance kit as per manufacturer's instructions. Digoxigenin-labeled locked nucleic acid (LNA)-oligos from Exiqon were hybridized at 50°C. Additional clones within the clonesets corresponding to *Pcdh7 *(clones H3060E02 and H3067F12) and *Pcdh17 *(clone AI843903) were used to confirm the observed pattern for these genes. Additional probes for other genes were produced by cloning various subregions: *Jagged1 *(nt716–1514 and 3747–4559), *Zcsl3 *(nt83–1004), *Gicerin *(nt23–1105, 904–2102, 1592–2249, 1993–2873), *CTGF *(nt1102–1697, 1686–2267), *Tbr1 *(nt521–1122 and nt2798–3398).

### Ablation

A solution of 5% zinc sulfate in PBS was injected using a 1 ml hypodermic syringe drawn out to produce a thin, flexible tube that can be easily inserted into the nares. Mice were anesthetized with isofluorane prior to instillation of 50–75 μl of zinc sulfate solution.

### Three-dimensional reconstruction

*In situ *hybridization data from E17.5 coronal sections were obtained for each gene every 60–80 μm along the AP extent of the bulb. The expression patterns for multiple bulbs were compared against one another, and were found to be similar. We therefore performed reconstructions using data from an individual bulb hybridized with any given probe. Images from hybridized sections were imported into Adobe Photoshop, and regions of high expression were selected and enhanced. These enhanced images were combined using ImageJ to create individual stacks of images corresponding to a given gene. A projection of the stacked images was then generated to produce a three-dimensional pattern of gene expression. These projections were then superimposed upon a cartoon depiction of the bulb to produce the final image.

## Authors' contributions

EOW and HS performed the *in situ *hybridization experiments. EOW performed the laser microdissection, microarray generation, microarray hybridizations, chemical ablations, and three-dimensional reconstructions. YY and JYHY did the statistical analysis. PS and GY performed computational analysis. DML performed the amplifications and wrote the manuscript with EOW. All authors have read and approved the final manuscript.

## Supplementary Material

Additional File 1 Experimental design and linear models. Click here for file
