# Supplementary material for: Novel subdomains of the mouse olfactory bulb defined by molecular heterogeneity in the nascent external plexiform and glomerular layers
Source: BMC Dev Biol. 2007 May 16;7:48. doi: 10.1186/1471-213X-7-48 (PMC1885806; doi:10.1186/1471-213X-7-48)
Supplement: Additional File 1 — Experimental design and linear models. [file 1471-213X-7-48-S1.pdf]

## Additional Files

Additional File 1
File format: PDF
Title: Experimental design and linear models
Description: This additional file describes in detail the experimental design and linear models used to analyze the microarray data.

In order to generate a three-dimensional representation of gene expression within the bulb, four LMD samples, corresponding to isolates from the DL, VL, DM and VM aspect of each section, were obtained from the 5$^{th}$, 6$^{th}$, 8$^{th}$, 16$^{th}$, 18$^{th}$, 22$^{nd}$, 26$^{th}$, 30$^{th}$ and 32$^{nd}$ sections along the AP axis. Expression data from these 36 regionally defined samples were combined and contrasted to generate the final representation. Given that these samples differ solely by location, we expected minimal differences in expression among the various LMD isolates. To identify these subtle changes in gene expression, we developed and tested several models. Each model was experimentally validated or eliminated by *in situ* hybridization. The experimental design and model comparison are detailed below, as are the final gene lists used in this study.

*Experimental Design*

We performed, in parallel, both indirect, “reference design” hybridizations as well as direct, loop design-based hybridizations. Each design format has its own advantages and disadvantages (Kerr and Churchill, 2001; Churchill, 2002; Glonek and Solomon, 2002; Yang and Speed, 2002). In the reference design, each LMD sample was hybridized against a common reference sample comprised of whole brain RNA (e.g. Fig. S1A). In the loop design, all four LMD samples from each section along the AP axis of the bulb were hybridized against one another (e.g. Fig. S1B). Samples across sections were not hybridized together in the loop design. In addition, the

direction of the loop was varied (clockwise or counterclockwise) to address any potential dye-effects that may be present.

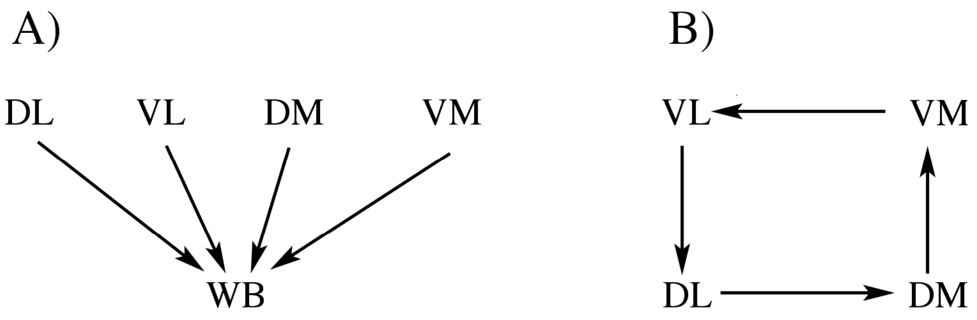


**Figure S1.** A) Example of common reference design. B) example of direct comparison design. VL, VM, DL and DM are the four LMD sections; WB refers to whole brain RNA. The arrow points from the Cy5 sample to the Cy3 sample.

A typical dataset from an individual hybridization using the common reference design is shown in Fig. S2. The MA-plot shows a large number of differences between the LMD sample and the reference. This invalidates most commonly used normalization approaches, where it is assumed the majority of the genes on the array are similar in expression between the two hybridized samples. To circumvent this problem, a within-slide median normalization was performed using internal controls spotted on the slide that have previously been shown to function as relatively invariant normalization controls (Yang et al., 2002). Despite this, a strong fan shaped pattern was still present, indicating that normalized values still varied significantly between the LMD and reference.

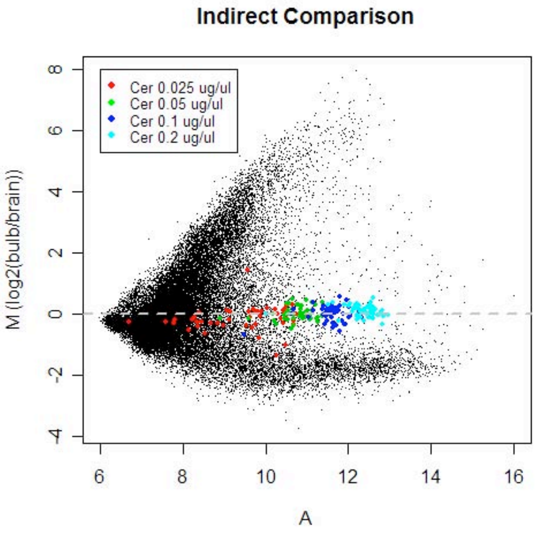


**Figure S2**. *MA*-plot of the common reference design with the normalization control titration series highlighted.

To better assess the utility of the common reference approach, data from LMD samples (e.g. 5VL and 5DL) that were directly hybridized against one another was compared against data obtained by hybridizing these same samples against a common reference (Figure S3). Shown in red are values from our internal, normalization controls. Shown in purple are genes that we determined in retrospect to represent true positives by verifications of *in situ* hybridization. The ability to detect such positives or to distinguish them from the general noise is significantly impaired in the indirect approach. The dramatic spread in values obtained with the indirect approach argues that, for this experiment, the common reference design is inherently more variable than the direct approach. This series of hybridizations was therefore excluded from further downstream analysis. We note that the inability to detect DE genes in the common reference design is likely due to the small effect size between different regions of the bulb. Other common references were also tested, with similar effect.

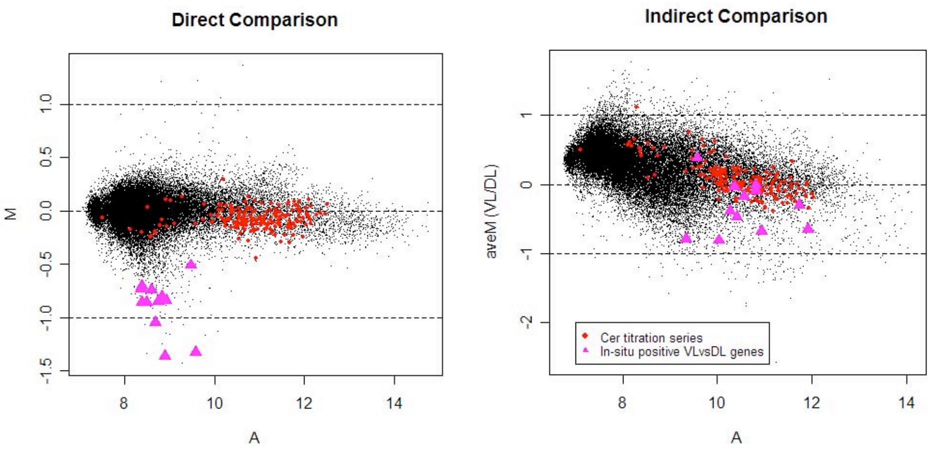


**Figure S3**. A typical comparison between direct (left) and indirect design (right). Titration control series and in situ positive genes are highlighted in red and purple respectively.

*Linear models comparisons*

As there are few natural approaches to analyze this data, we developed three different models. To determine which model would best represent the data, a subset of genes from each model was tested by *in situ* hybridization. Based on this empirical data, the individual model(s) were further modified and re-tested.

Model 1

We first developed a model that represented the spatial information present in the array data. We used a linear model with two factors representing the DV and ML axes of the olfactory bulb. As the loop design did not incorporate hybridizations across sections, we essentially treated all data obtained from any point along the AP axis as biological replicates. For a given gene, the model is written as

$$M_{abk} = \mu + \alpha_a + \beta_b + \alpha\beta_{ab} + \varepsilon_{abk}$$

where *a=b=1,2*. In this 2 x 2 factorial (or two-way ANOVA) model, the parameter $\mu$ is a baseline effect; $\alpha$ and $\beta$ denote the main effects from the DV-axis and ML-axis respectively and $\alpha\beta$ denotes the 2-way interaction effects between the DV and ML axes. The intensity log-ratio $M$ is the observed value for a particular gene and the random variable $\varepsilon$ is an error term assumed to be distributed with a mean of 0 and constant variance $\sigma^2$ independent for each slide. This model can easily be interpreted in light of our predicted spatial expression patterns. For genes with a significant 2-way interaction effect (i.e. extreme $\alpha\beta$ values), this class of genes would represent expression cues that have a more localized pattern. Genes with no 2-way interactions effects but significant main effects ($\alpha\beta = 0$) would be predicted to be expressed in a DV or ML axis in a gradient.

<u>Model 2</u>

Our second proposed approach of this experiment was to use a linear model to estimate the four main comparisons of interest. To estimate the spatial gene expression for a typical gene *g,* we treat all the spatial sections along the AP-axis as replicates and fit the following linear model:

$$M_{ik} = \alpha_i + \varepsilon_{ik}$$

where $i = 1,..,4$ represents the four main comparisons of interest; $M_{ik}$ is a vector of log-ratios and $\mathrm{var}(\varepsilon_{ik}) = \sigma$. The parameter $\alpha_i$ can be estimated by the normal equation. In practice, the data were fitted to the linear model above based on the least square procedure using the function *lmFit* provided in the library *limma* (Smyth, 2004) in the statistical software package R. Estimates of the coefficient as well as the corresponding moderated *t*-statistics (Smyth, 2004) and adjusted p-values ware calculated.

Model 3

In the third model, for a typical gene *g*, the intensity value corresponding to the four different sub-regions of the bulb is denoted by $DM_k, DL_k, VM_k, VL_k$, where k represents the different sections taken along the AP-axis. The log transformation of these values is defined as $dm_k = \log_2 DM_k$ , $dl_k = \log_2 DL_k$ , $vm_k = \log_2 VM_k$ and $vl_k = \log_2 VL_k$ . To estimate the spatial gene expression for gene *g,* all of the spatial sections along the AP-axis are treated as replicates and used to fit four separate linear models for each of the comparisons of interest $\alpha_1 = dm - dl,$ $\alpha_2 = dm - vm,$ $\alpha_3 = vl - dl$ and $\alpha_4 = vm - vl$ . The linear model is written as

$$M_{ik} = \alpha_i + \varepsilon_{ik}$$

where $i = 1,..,4$ representing the four main comparisons of interest; $M_{ik}$ is a vector of log-ratios and $\mathrm{var}(\varepsilon_{ik}) = \sigma_i$ . This model is equivalent of performing four separate series of one-sample comparisons on four separate sets of data. Estimates of the coefficient of variation as well as the corresponding moderated *t*-statistics and adjusted p-values were calculated using the function *lmFit* provided in the library *limma* (Smyth, 2004) in the statistical software package R.

As essentially no differentially expressed genes had been identified within the EPL prior to this analysis, we arbitrarily chose to validate the three models focusing on the $vl_g - dl_g$ comparisons. We reasoned that gene expression differences along the DV axis were likely to exist based upon the known, zone-to-zone innervation of the bulb by OSNs. However, no prior empirical proof of this regarding confirmed genes was available. For each of the 3 proposed models, the top 10 genes based on the smallest adjusted p-values were selected for the initial round of *in situ* validation. For the three models, 80, 60, and 100%, respectively, were proven to be spatially differential, with model 3 providing the highest number of positive hits.

The validation results were rather unexpected. Although all were successful, in theory, we expected model 1 to provide the best interpretation of the spatial representation of the data. However the relatively lower validation rate could be due to the limited spatial resolution in this current data set. The key difference between model 1, which was developed first, and the other two models was an attempt at the use of both direct and indirect comparisons in the estimation of $vl_g - dl_g$. The main difference between models 2 and 3 is in the estimation of variance for each gene. The random variable $\varepsilon$ is an error term assumed to be distributed with a mean of 0 and constant variance $\sigma^2$ in model 2 but assumed to be distributed with variance $\sigma_i^2$ in model 3. In other words, we used the pooled variance estimates from all 4 comparisons in model 2, in contrast, model 3 only uses variance estimates from a subset of arrays corresponding to a specific comparison.

*References*


Churchill GA (2002) Fundamentals of experimental design for cDNA microarrays. Nat Genet 32 Suppl:490-495.

Glonek GFP, Solomon PJ (2002) Factorial and time course designs for cDNA microarray experiments. Technical Report, Department of Applied Mathematics University of Adelaide.

Kerr MK, Churchill GA (2001) Experimental design for gene expression microarrays. Biostatistics 2:183-201.

Smyth GK (2004) Linear models and empirical Bayes methods for assessing differential expression in microarray experiments. Statistical Applications in Genetics and Molecular Biology 3:Article 3.

Yang YH, Speed T (2002) Design issues for cDNA microarray experiments. Nat Rev Genet 3:579-588.

Yang YH, Dudoit S, Luu P, Lin DM, Peng V, Ngai J, Speed TP (2002) Normalization for cDNA microarray data: a robust composite method addressing single and multiple slide systematic variation. Nucleic Acids Res 30:e15.

## Supplemental Material: Table 1.

### DL vs. DM (33%)

| | |
|---|---|
| **H3030C10** | |
| H3008H11 | |
| H3026C10 | Homo sapiens zinc finger protein ANC_2H01 (LOC51193), mRNA |
| AI838694 | ESTs |
| **H3064C05** | |
| H3026F08 | Mus musculus Ccth gene for chaperonin containing TCP-1 eta subunit, complete cds |
| H3059B03 | Mus musculus protein kinase C delta mRNA, complete cds |
| **H3098F08** | |
| H3017D11 | Homo sapiens cDNA: FLJ21762 fis, clone COLF6920 |
| H3028F11 | |
| **H3118G08** | Mus musculus MHC class III region RD gene, partial cds; |
| H3146B04 | |

### VL vs. DL (83%)

| | |
|---|---|
| **H3123A06** | |
| **H3099B10** | |
| **H3052H04** | Mus domesticus strain MilP mitocondrion genome, complete sequence |
| **H3101G12** | |
| **H3086G04** | |
| **H3045F09** | |
| **H3109A11** | |
| **H4068H06** | UNKNOWN |
| **H3081D05** | |
| **H3033D03** | |
| **H3097H02** | |
| H3091C06 | |
| H3045F07 | Mus musculus hypothetical brain protein similar to X96994 BR-1 protein (Helix pomatia) |

### VM vs. VL (8%)

| | |
|---|---|
| H4072A08 | UNKNOWN |
| AI845459 | Not available at this time |
| H4033A05 | UNKNOWN |
| **H3005G10** | |
| H3148G11 | |
| H3102H09 | Mus musculus ribonuclease H1 (Rnaseh1), mRNA |
| H4055B08 | UNKNOWN: Similar to Homo sapiens similar to CG3570 gene product (LOC154743), mRNA |
| AI851792 | ESTs, Weakly similar to rhophilin [M.musculus] |
| H3057C01 | Mus musculus interferon-related developmental regulator 1 (Ifrd1), mRNA |
| H3151E01 | Homo sapiens mRNA; cDNA DKFZp434M232 (from clone DKFZp434M232) |
| H4061C03 | Mus musculus similar to ISS~putative~reduced expression 2 (LOC213580), mRNA |
| H3142G03 | Homo sapiens cDNA: FLJ21288 fis, clone COL01927 |
| H3142G06 | Mus musculus cadherin 11 (Cdh11) gene, exon 3 |

### DM vs. VM (33%)

| | |
|---|---|
| **AI847909** | ESTs, Weakly similar to p58 [M.musculus] |
| H3157F06 | Mus musculus growth associated protein 43 (Gap43), mRNA |

| | |
|---|---|
| **H3144G05** | Homo sapiens protocadherin 68 (PCH68), mRNA |
| **H4051A04** | |
| H3147E03 | Mus musculus Stat3ip1-pending mRNA |
| AI845459 | Not available at this time |
| AI844744 | Mus musculus G protein signaling regulator RGS2 (rgs2) mRNA, complete cds |
| H4052A04 | Mus musculus sialyltransferase 4A mRNA |
| **H3146E06** | Mus musculus mRNA for s-gicerin/MUC18, complete cds |
| H3046D09 | Mus musculus ribosomal protein S6 kinase polypeptide 1 (Rps6ka1), mRNA |
| H3124A09 | Mus musculus MAP kinase kinase 7 gamma 2 mRNA, complete cds |
| H4038G08 | |
| Additional | |
| **H4017A09** | UNKNOWN: Similar to Rattus norvegicus jagged 1 (Jag1), mRNA |
| **H4067E03** | Mus musculus connective tissue growth factor (Ctgf), mRNA |
| **H3116C09** | Mus musculus protocadherin 7 (Pcdh7), mRNA |

**Table 1. Predicted cDNAs tested for spatial differential expression.** Predicted differentially expressed genes were placed into four groups (DL vs. DM, VL vs. DL, VM vs. VL, and DM vs. VM) based on the linear model. From each group, we selected the top 12 cDNAs to test for spatial differential expression by *in situ* hybridization. Those in bold were confirmed as being differentially expressed. The percentage within each category are listed at the top of each group heading. In addition, we picked a subset of genes for testing that were further down each individual group based on their annotation. However, these genes were still within the top 50 by rank. These are listed under the heading Additional.
